# Resource-use efficiency explains grassy weed invasion in a low-resource savanna in north Australia

**DOI:** 10.3389/fpls.2015.00560

**Published:** 2015-08-04

**Authors:** Emilie Ens, Lindsay B. Hutley, Natalie A. Rossiter-Rachor, Michael M. Douglas, Samantha A. Setterfield

**Affiliations:** ^1^Research Institute for the Environment and Livelihoods, Charles Darwin University, Darwin, NTAustralia; ^2^Department of Environmental Sciences, Macquarie University, North Ryde, NSWAustralia; ^3^School of Earth and Environment, University of Western Australia, Perth, WAAustralia

**Keywords:** alien invasive species, ecophysiology, water use, carbon uptake, weed invasion, trait-based comparisons, stable isotopes, carbon

## Abstract

Comparative studies of plant resource use and ecophysiological traits of invasive and native resident plant species can elucidate mechanisms of invasion success and ecosystem impacts. In the seasonal tropics of north Australia, the alien C_4_ perennial grass *Andropogon gayanus* (gamba grass) has transformed diverse, mixed tree-grass savanna ecosystems into dense monocultures. To better understand the mechanisms of invasion, we compared resource acquisition and usage efficiency using leaf-scale ecophysiological and stand-scale growth traits of *A. gayanus* with a co-habiting native C_4_ perennial grass *Alloteropsis semialata*. Under wet season conditions, *A. gayanus* had higher rates of stomatal conductance, assimilation, and water use, plus a longer daily assimilation period than the native species *A. semialata.* Growing season length was also ~2 months longer for the invader. Wet season measures of leaf scale water use efficiency (WUE) and light use efficiency (LUE) did not differ between the two species, although photosynthetic nitrogen use efficiency (PNUE) was significantly higher in *A. gayanus*. By May (dry season) the drought avoiding native species *A. semialata* had senesced. In contrast, rates of *A. gayanus* gas exchange was maintained into the dry season, albeit at lower rates that the wet season, but at higher WUE and PNUE, evidence of significant physiological plasticity. High PNUE and leaf ^15^N isotope values suggested that *A. gayanus* was also capable of preferential uptake of soil ammonium, with utilization occurring into the dry season. High PNUE and fire tolerance in an *N*-limited and highly flammable ecosystem confers a significant competitive advantage over native grass species and a broader niche width. As a result *A. gayanus* is rapidly spreading across north Australia with significant consequences for biodiversity and carbon and retention.

## Introduction

Alien plant invasions are considered a major threat globally to biodiversity and ecosystem function (Simberloff, 2011; Vilà et al., 2011; Strayer, 2012). Considerable research effort has gone into understanding the mechanisms that drive invasion success in order to direct effective weed management activities (Blumenthal, 2006; Barney and Whitlow, 2008; Catford et al., 2009). Invasion drivers vary and are mediated or filtered by characteristics of the ecosystem being invaded, which can also differ in space and time (D’Antonio, 1993; Levine et al., 2003; Theoharides and Dukes, 2007). One of the major drivers of successful invasion is resource competition (Levine et al., 2003; Vilà and Weiner, 2004). Successful invaders are typically considered to possess a superior ability to acquire limiting resources (e.g., light, nutrients), and/or allocate resources to different plant parts for improved performance (Goldberg et al., 1999). Generally, high resource environments tend to be more invasible than low-resource environments (Gross et al., 2005; Funk, 2013); native species are considered more likely to have a competitive advantage over alien plants in low-resource environments (Funk, 2013). However, in a major review on this topic, Gioria and Osborne (2014) found few studies that compared resource competition directly and most studies were undertaken in high resource environments. Many studies were also confounded by factors such as comparisons of different life forms or dominant alien versus subordinate native species. The effects on carbon sequestration and water use when species replacement is by another of the same life form will depend largely on individual species attributes and climate and may be difficult to predict (Cavaleri and Sack, 2010).

This study focuses on the mechanisms facilitating the invasion of C_4_ *Andropogon gayanus* Kunth. (gamba grass) in Australia’s mesic (>900 mm annual rainfall) savannas. Large areas (>200,000 ha) of invasion are occurring across the ‘Koolpinyah surface’ (Nott, 1995), a regional geomorphological formation that consists of ancient (Late Tertiary), leached, undulating sandy plains of low soil N and low organic carbon (Scott et al., 2009; Smith and Hill, 2011). Savanna ecosystems being invaded can be considered a resource-limited ecosystem due to these low fertility soils coupled with annual drought (6 months per year) and frequent fire (2 in 3 years) (Hutley and Setterfield, 2008). Despite the limiting resources, *A. gayanus* is one of a number of introduced pasture species that have become successful invaders in this region (Cook and Dias, 2006; Setterfield et al., 2013). Some drivers of *A. gayanus* invasion success have been previously demonstrated. For example, *A. gayanus* produces large amounts of seed annually compared to native grasses (Flores et al., 2005; Setterfield et al., 2005), resulting in high propagule pressure typical of successful invaders (Eppstein and Molofsky, 2007; Catford et al., 2009). Seedling establishment occurs in intact savanna but is greatly facilitated by both canopy cover and/or ground layer disturbance (Setterfield et al., 2005). Like many successful invaders, *A. gayanus* alters the abiotic characteristics of invaded sites to enhance its ability to colonize and survive (Catford et al., 2009). In this situation, the dominant fire regime changes as a consequence of the increased *A. gayanus* derived fuel loads and fire intensity (Rossiter et al., 2003; Setterfield et al., 2010) resulting in reduced canopy cover and ground layer vegetation and increased site suitability for establishment of the invader (Rossiter et al., 2003; Setterfield et al., 2005). These drivers contribute to the initial invasion of *A. gayanus* but the rapid establishment and expansion of this species is likely to be due to other mechanisms that allow the alien species to have competitive advantages over the native species in this low-resource environment.

Studies examining invasion by C_4_ grass into low-resource environments suggests the importance of understanding ecophysiological differences between the invaders and native species (Chapin et al., 1996; Williams and Baruch, 2000; Daehler, 2003). In South America’s neotropical savannas, the higher maximum stomatal conductance, photosynthesis, and transpiration rates of two invasive C_4_ grasses compared to the dominant native C_4_ grasses were suggested as partially explaining their invasion success (Baruch and Fernandez, 1993; Baruch and Gomez, 1996). Similarly, in Hawaii, the invasion of alien C_4_ *Pennisetum setaceum* (Forsk.) Chiov. was partially attributed to high maximum photosynthetic rates compared to the native C_4_ *Heteropogon contortus* (Williams and Black, 1994). Despite this competitive advantage, in both of these studies, the native grass was found to have a greater tolerance to soil water deficit and the growth of the alien grass was constrained by water availability (Baruch and Fernandez, 1993; Williams and Black, 1994). This would limit the spatial distribution and growing season of the alien C_4_ grasses, providing insights into how to control these species and restore the ecosystem (Funk, 2013). At present it is uncertain what constraints may limit the spread of *A. gayanus* and this study provides further assessment of the likely ecophysiological mechanisms and their importance driving the replacement of a resident native C_4_ grass flora by an alien and invasive C_4_ grass. We compared 13 ecophysiological and growth traits of the alien *A. gayanus* and native *Alloteropsis semialata* (R. Br.) Hitchc. In particular, we investigated the (1) diurnal and seasonal patterns of leaf gas exchange and stomatal conductance, (2) maximum photosynthesis and transpiration rates under saturating radiation, (3) photosynthetic responses to leaf to air vapour pressure difference (LAVPD), (4) leaf scale efficiencies of light, water and nitrogen use, (5) canopy scale carbon and water fluxes, (6) foliar nitrogen, and (7) foliar C and N isotopes.

## Materials and Methods

### Study Location

The study was undertaken at Mary River National Park (formerly Wildman Reserve; 12°43′S, 131°49′E), Northern Territory, Australia. The savanna vegetation at the site is dominated by canopy *Eucalyptus miniata* (Cunn. Ex Schauer) and *E. tetrodonta* (F. Muell) with a cover of 40–50% and a canopy height of 15–20 m. This vegetation assemblage occupies approximately 246, 600 km^2^ across Australia’s savanna region (Fox et al., 2001). The climate is characterized by distinct wet season (October–March) and dry seasons (May–September), the latter of which has high vapor pressure deficits (VPD, 2–5 kPa; Egan and Williams, 1996). Mean annual rainfall at Mary River National Park is 1433 mm and mean annual temperature is 27°C (Commonwealth Bureau of Meteorology). Soil types at Mary River National Park are sandy loam red and gray Kandosols (after Isbell, 1996) that are characterized by low nutrient levels with a soil organic carbon content (<2%) and low nitrogen content in the surface horizons of from 0.01 to 0.11% (Day et al., 1979; Rossiter-Rachor, 2008). These soils are coarse textured and well drained, but with low water holding capacity.

**FIGURE 1:**
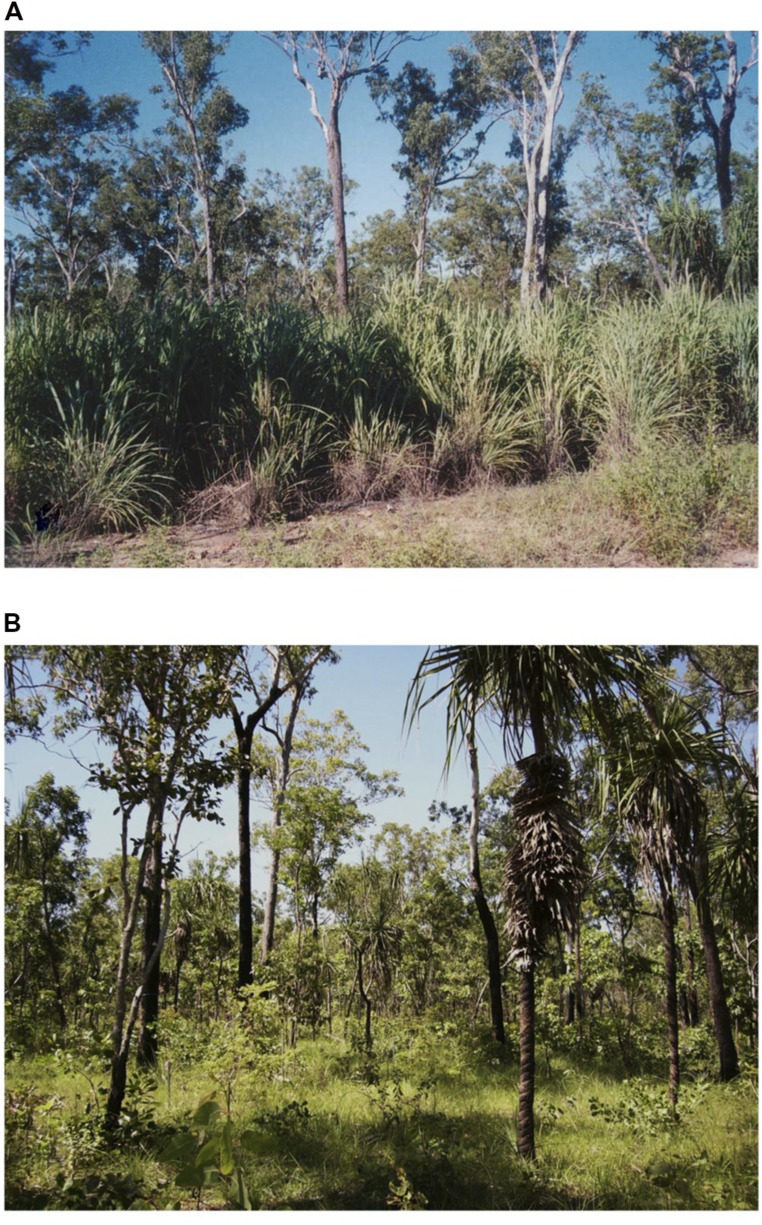
**(A)** Tropical savanna invaded with *A. gayanus* at Mary River National Park, Northern Territory, Australia. The alien grass forms extensive blocks with a sharp invasion front adjacent to **(B)** uninvaded savanna blocks with an understorey of the native C4 grass *A. semialata*, as used in this study. (Photo credits *N. Rossiter-Rachor*).

The native grass understorey consists of perennial C_4_ grasses such as *Alloteropsis semialata, Heteropogon triticeus, Chrysopogon fallax, Eriachne triseta* Nees ex Steud., and C_4_ annual grasses such as *Pseudopogonatherum irritans* (Br.) and *Sorghum* sp. Following the release of a commercial seed supply in 1983, the alien grass *A. gayanus* (cultivar ‘Kent’) was planted at a range of locations across northern Australia (Oram, 1987) including near Mary River National Park and has since invaded vast areas of the high rainfall savanna (>1000 mm annual rainfall; Petty et al., 2012; Adams and Setterfield, 2013). It is now a dominant feature of the understorey in the northern section of the national park. *A. gayanus* can form dense monospecific sward up to 4 m high with a biomass of 20–30 t ha^-1^ in heavily invaded patches (Rossiter et al., 2003), with a sharp invasion front adjacent to non-invaded savanna (**Figures 1A,B**). Comparisons were undertaken at paired non-invaded, and invaded sites. Invaded sites had a minimum of 70% cover of *A. gayanus* in the understorey, whereas non-invaded sites had no *A. gayanus* and were dominated by *A. semialata*.

### Leaf Gas Exchange

Leaf scale physiological traits of *A. gayanus* and *A. semialata* were compared using two approaches. Firstly, observations of diurnal patterns of leaf gas exchange were tracked for the two species using plants from three plots-pairs (*A. gayanus* vs native grasses) within the Mary River National Park. Measurements were made *in situ* during the wet (March) and dry seasons (May) using a portable photosynthesis system (Li-Cor 6400, Li-Cor Inc., Lincoln, NE, USA) on plants within adjacent sward of *A. gayanus* and *A. semialata* across an invasion front (**Figures 1A,B**). Ambient conditions were maintained within the leaf chamber with the instrument in standard measurements mode. Care was taken to ensure the exposure to incident radiation to a leaf was maintained during measurements. Afternoon air temperatures reached 35°C and heating of the instrument occurred requiring regulation of the chamber temperature which was set to 35°C to prevent artificial warming of leaves during measurement.

Native grasses were not measured in the dry season as leaves had senesced by this phase of the seasonal cycle, whereas *A. gayanus* plants still supported green foliage enabling wet and dry season comparisons. Dry season measurements for *A. gayanus* were at the same site using the same population of plants and leaves. These diurnal gas exchange measurements provided *in situ* measurements of leaf performance over a range of leaf and air temperature and light conditions. Secondly, a further set of observations were made during the wet season (March) at an additional three sites within the Mary River National Park. This was undertaken to examine spatial variation of maximum net photosynthesis (A_max_) and transpiration (T_max_) of both species under conditions of saturating light. Again, *A. gayanus* and *A. semialata* were sampled across an invasion front at these additional sites.

Gas exchange measurements were made on fully expanded, mature leaves approximately two thirds along the leaf lamina of five randomly selected individual plants. Measurements were made on three leaves per *A. gayanus* plant and two leaves per *A. semialata* plant, given the small plant and leaf size of the latter. This provided a total of 25 leaves sampled across both species per sampling run, which took approximately 1 h to complete. This sampling cycle was repeated continuously from 1000 to 1700 h local time. Variables collected per leaf included leaf temperature (T_leaf_), leaf to air vapour pressure deficit (LAVPD), photosynthetically-active flux density incident at the leaf surface (PAR), assimilation (A), transpiration (T), and stomatal conductance (g_s_).

Gas exchange measurements were made at three additional sites under saturating light conditions with measurements occurring between 11 am and 1500 h local time. These measures were used for the analysis of instantaneous transpiration efficiency (ITE) and intrinsic water use efficiency (IWUE). ITE was calculated as μmol of CO_2_ assimilated per mol of water transpired (A/T). IWUE was defined as the ratio of light saturated net assimilation rate to stomatal conductance (A/g_s_) which is thought to have low dependence on environmental parameters and reflects intrinsic plant physiological functioning (Jones, 1992). To determine the light compensation points, the light saturation point and apparent quantum yields light use efficiency, (LUE; Kuppers and Schulze, 1985), non-linear regressions (A = a × ln(PAR) - b) were fitted to the PAR and A data for each species. Instantaneous LUE of each species was quantified as μmol of CO_2_ assimilated per μmol^-1^ PAR in the light limited region of the light response curves.

### Above-Ground Biomass and Leaf Area Index

In March and May 2003, the above-ground live plant material (leaves and stems) of each species was harvested in three random 2 m × 2 m quadrats at each of the three paired invaded and non-invaded sites used in the gas exchange measurements. Plant material was dried and weighed to give above-ground biomass (AGB) and scaled to leaf area using allometric equations developed for *A. gayanus* (Rossiter, 2001). A generic allometric equation for native grass biomass and leaf area was used as a surrogate for *A. semialata* as this relationship has been shown to hold across a range of northern Australian tropical savanna C_4_ grasses (Hutley and Williams, unpublished data). Native grass species used to derive the allometric equations were all C_4_ grasses common in these tropical savanna woodlands (Scott et al., 2012) and included *Aristida hygrometrica*, *Chrysopogon latifolius, Sorghum intrans, Heteropogon triticeus, Themeda triandra, Sehima nervosum, Sorghum plumosum, Chrysopogon fallax, Setaria apiculata, Pseudopogonatherum contortum* variously from four sites (Howard Springs, Claravale, Larrimah, and Katherine). Estimates of leaf area sampled from the 2 m × 2 m area enabled the mean leaf area index (LAI) for each site (invaded or non-invaded) to be estimated.

### Leaf Nitrogen, Carbon, and Isotopes

All leaves used in the gas exchange measurements of both species were collected, dried, pooled, and ground in a Culatti Type grinder (Model MFC CZ13) with a 1 mm screen. Percent elemental carbon (%C) and nitrogen (%N) and stable carbon (δ^13^C) and nitrogen (δ^15^N) isotope ratios were determined via Dumas combustion in an IsoChrom which was connected to an EA-1110 Elemental CHN-O Analyser. Analysis was conducted by the Australian National University stable isotope facility. Foliar nitrogen values (g N/g leaf) were divided by A_max_ values for each leaf to calculate photosynthetic nitrogen use efficiency (PNUE). Foliar δ^13^C values indicate long term water use efficiency (LTWUE, Dawson et al., 2002). Foliar leaf δ^15^N values are somewhat indicative of nitrogen source and soil availability, with lower values suggesting preferential uptake of ammonium or higher soil ammonium to nitrate ratios (Handley et al., 1998).

### Statistical Analysis

All statistical analyses were performed using SPSS Version 6.0 (2007, SPSS Inc., Chicago, IL, USA). Clear outliers (>2 SD from the mean) were removed prior to analysis. Outliers were clearly identified from raw data plots and data points that were approximately 2 SD from a measurement run mean were examined. In the wet season, the percentage of outliers was larger than 5% and was 11% for *A. gayanus* and 10% for *A. semialata*. None were identified for the *A. gayanus* dry season data set. Variable PAR conditions and shifts in temperature and VPD with cloud cover of the wet season resulted in a population of leaves that were not at a stable equilibrium when measured and were not included in calculations.

Diurnal leaf temperature, PAR, LAVPD, A, T, and gs, at each time period (10:00, 11:00, 13:00, 14:00, and 16:00 or 17:00) were analyzed using a one-way ANOVA to compare differences between the species/season factor (fixed). Data was normal (skewness, kurtosis values <2) and variances homogeneous (Levene’s significance test <0.05). Data for diurnal analyses were based on plants at one paired site only. Differences between species and seasons were assessed using the Student-Neumann–Keuls *post hoc* test. The species/season factor included three variables: *A. gayanus* in the wet season, *A. semialata* in the wet season, and *A. gayanus* in the dry season.

Estimates of daily carbon uptake rates and water use per ground area (canopy scale fluxes) were determined by scaling up integrated diurnal measures of A and T, respectively, to rates per square meter ground area using site based LAI estimates. Extrapolation of leaf gas exchange parameters to the canopy scale using LAI is based on the assumption that for these grasses, canopy self-shading is limited and simple scaling using LAI to obtain canopy level estimates is feasible (Larcher, 2003). Leaf scale estimates and foliar δ^13^C, δ^15^N, foliar %N, foliar %C, foliar C:N, and PNUE (A_max_/*N* per g leaf, PNUE) for each species/season were analyzed using one-way ANOVA and Student-Neumann–Keuls *post hoc* tests to assess differences between species and season (for *A. gayanus* data). Differences in ITE (A/T) and IWUE (A/g_s_) for each species/season (all sites data) were analyzed using a one-way ANOVA, Student-Neumann–Keuls *post hoc* tests and pair-wise comparisons with a Bonferroni adjustment.

The LUE of each species was determined as the slope of the linear relationship between PAR and A when light was limiting. Light limitation was assumed to have occurred at PAR <480 μmol m^-2^ s^-1^. Differences were analyzed using an ANCOVA with PAR as the covariate and species/season as the fixed factor. Differences in A_max_, T_max_, and g_s_ for each species were determined from the light saturated leaves when PAR >750 μmol m^-2^ s^-1^ for *A. gayanus* and *A. semialata* in the wet season and PAR >500 μmol m^-2^ s^-1^ for *A. gayanus* in the dry season. Differences were compared using ANCOVA, with PAR as the covariate and species/season as the fixed factor.

## Results

### Leaf Microclimate

**FIGURE 2:**
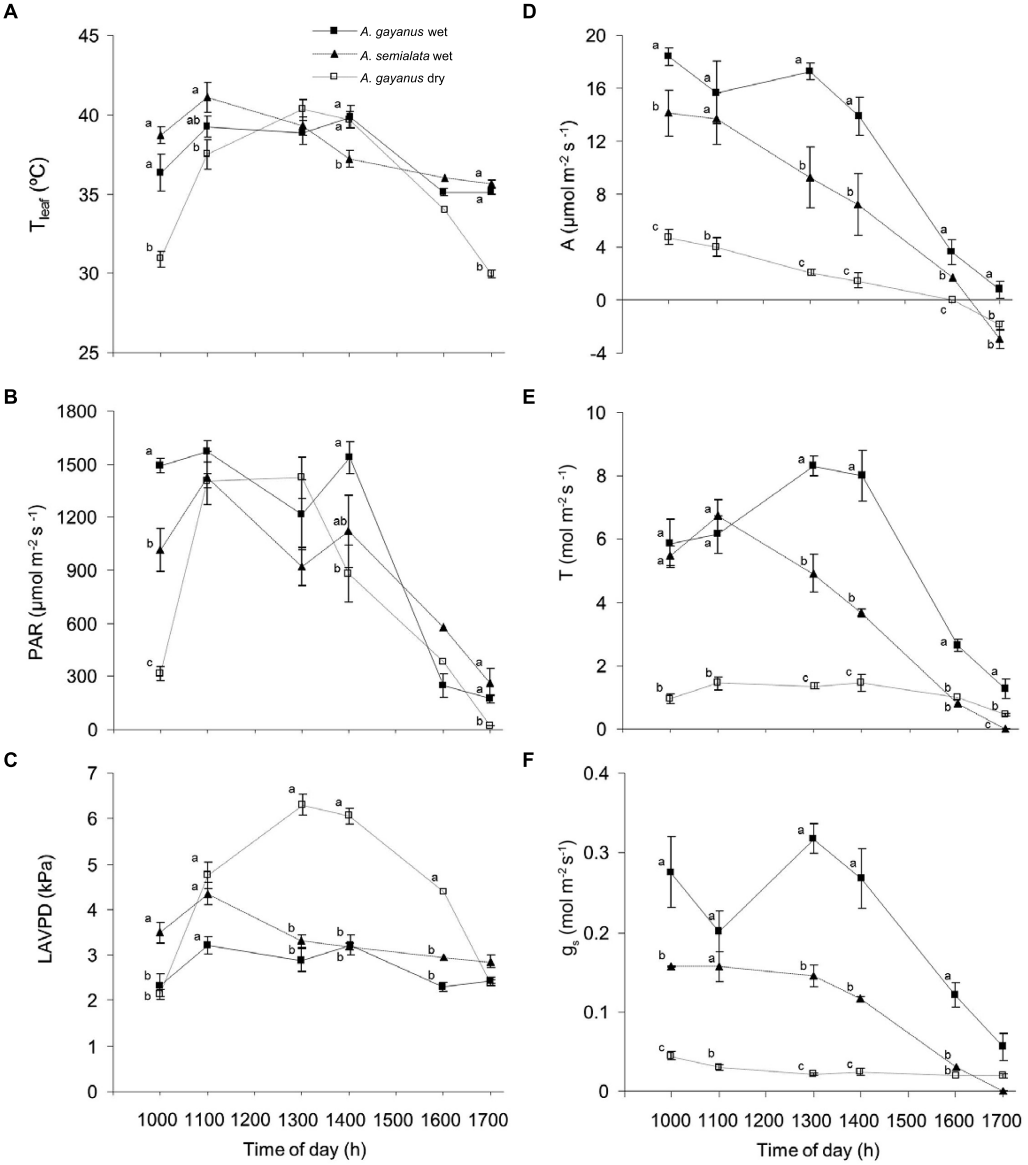
**Diurnal patterns of leaf scale **(A)** temperature (T_leaf_), **(B)** photosynthetically active flux density incident at the leaf surface (PAR), **(C)** leaf to air vapor pressure difference (LAVPD), **(D)** assimilation (A), **(E)** transpiration (T) and **(F)** stomatal conductance (g_s_) of *A. gayanus* in the wet (closed squares, *n* = 120), *A. semialata* in the wet (triangles, *n* = 120), and *A. gayanus* in the dry (open squares, *n* = 120) seasons.** Error bars represent one standard error of the mean. Different letters at each time indicate significant differences at *P* = 0.05.

During the wet season, *A. gayanus* and *A. semialata* leaves experienced a broadly similar microclimate in terms of T_leaf_, leaf incident PAR and LAVPD, enabling direct species comparisons of physiological variables (**Figure 2A**, T, g_s_.). Leaf temperatures of both species were similar throughout the day, although at approximately 1400 h T_leaf_ of *A. gayanus* was higher than that of *A. semialata* (**Figure 2A**). Levels of PAR were also similar for both species except at 1000 h, when PAR was significantly higher for *A. gayanus* (**Figure 2B**). The diurnal range of LAVPD of *A. semialata* leaves was similar to that of *A. gayanus* from 1100 to 1600 h, although leaves of *A. gayanus* had significantly higher LAVPD in the morning and afternoon (**Figure 2C**). By the dry season, early morning, and late afternoon T_leaf_ and PAR were significantly lower for the persistent *A. gayanus* compared to measurements in the wet season (**Figures 2A,B**). The LAVPD of *A gayanus* in the dry season was significantly higher than wet season measurements from 1100 to 1600 h (**Figure 2C**).

### Leaf and Canopy Scale Physiology

Both species showed similar decreasing linear trends in A throughout the day; however, in the wet season *A. gayanus* assimilated carbon at significantly higher rates than *A. semialata* (**Figure 2D**). Mean wet season rates of *A. gayanus* A_max_ and T_max_ were 30% higher than *A. semialata* (**Table 1**), with *A. gayanus* maintaining a longer daily period of assimilation compared to *A. semialata*. *A. semialata* exhibited net respiration by 1600 h while A remained positive for *A. gayanus* leaves until 1700 h (**Figure 2D**). Although lower than wet season rates, leaves of *A. gayanus*, were still assimilating carbon and transpiring in the dry season (**Figures 2D,E**) whereas *A. semialata* was physiologically dormant. In the wet season, morning levels of T and g_s_ for *A. gayanus* and *A. semialata* leaves were similar, however, by 1300 h, *A. gayanus* had significantly higher rates than *A. semialata. A. gayanus* was still transpiring water at 1700 h by which time *A. semialata* rates of T and g_s_ was close to zero (**Figures 2E,F**. Wet season gas exchange (*A*, *T*) was largely driven by PAR (**Figures 2B,D,E**).

**Table 1:** Mean eco-physiological traits of *A. gayanus* and *A. semialata* in the wet season, and *A. gayanus* in the dry season.

Species/season	AGB (g m^-2^)	LAI (m^2^ m^-2^)	A_max_ (μmol m^-2^ s^-1^)	T_max_ (mmol m^-2^ s^-1^)	g_s_ (mol m^-2^ s^-1^)	C uptake (g m^2^ d^-1^)	E (L m^2^ d^-1^)	ITE (A/T)	LUE (A/PAR)	PNUE (N/A)
**(a)**			
*A. gayanus*/Wet	183.9^a^ (11.78)	0.82 (0.04)	18.31 (0.51)	6.35 (0.17)	0.27^a^ (0.01)	2.68^a^ (0.08)	2.51^a^ (0.06)	1.73^a^ (0.29)	0.017^a^ (0.002)	0.06^a^ (0.003)
*A. semialata*/Wet	44.0^b^ (1.77)	0.22 (0.01)	11.81^b^ (0.55)	4.86^b^ (0.25)	0.16^b^ (0.05)	0.54^b^ (0.08)	0.40^b^ (0.06)	2.37^ab^ (0.09)	0.022^a^ (0.002)	0.10^b^ (0.01)
*A. gayanus*/Dry	405.5^c^ (58.86)	1.11 (0.06)	3.31^c^ (0.29)	1.42^c^ (0.07)	0.03^c^ (0.002)	0.97^b^ (0.12)	0.70^c^ (0.08)	2.53^b^ (0.22)	0.008^b^ (0.003)	0.31^c^ (0.05)
**(b)**			
*A. gayanus*/Wet	15	15	94	94	94	3	3	210	18	210
*A. semialata*/Wet	15	15	59	59	59	3	3	108	8	108
*A. gayanus*/Dry	15	15	71	71	71	3	3	120	15	120

*^a^Mean (SE) are given for above-ground biomass (AGB), leaf area index (LAI), maximum net photosynthesis (*A_max_*), maximum transpiration (*T_max_*), stomatal conductance (*g*_s_), carbon uptake (C uptake), evaporation (E), instantaneous transpiration efficiency (ITE), instantaneous light use efficiency (LUE), photosynthetic nitrogen use efficiency (PNUE). Different superscripts in each column indicate significant differences at *P* = 0.05. ^b^Number of samples used to derive all variables.*

Leaf gas exchange rates were extrapolated to a canopy level using LAI estimates to provide mean daily A and T per unit ground area. In the wet season, *A. gayanus* stands assimilated ~5 times more C per day and transpired six times more water than *A. semialata* (**Table 1**). Stand scale *A. gayanus* assimilation and water use in the dry season was still double that of the wet season rates of *A. semialata*, although this difference was not significant (**Table 1**).

### Foliar N, C, and Isotopic Signatures

There was no significant difference between foliar %N, %C, or δ^13^C for leaves of both species and nor between seasons for *A. gayanus* (**Table 2**). *A. semialata* and *A. gayanus* foliar δ^15^N values were similar in the wet season but were significantly lower than dry season values for *A. gayanus* (**Table 2**).

**Table 2:** Mean (SE) and ANOVA results for foliar percentage and isotopic nitrogen and carbon (%) of *A. gayanus (n* = 210) and *A. semialata* (*n* = 108) in the wet season and *A. gayanus* in dry season (*n* = 120).

Variable	*A. gayanus* wet season	*A. semialata* wet season	*A. gayanus* dry season	ANOVA
% foliar N	1.1. (0.08)^a^	1.14 (0.18)^a^	0.99 (0.15)^a^	*F*_(2,10)_ = 3.78; *P* = 0.695
% foliar C	46.44 (0.10)^a^	45.46 (0.39)^a^	45.93 (0.27)^a^	*F*_(2,10)_ = 3.64; *P* = 0.065
% foliar C: N	42.92 (2.92)^a^	42.24 (7.53)^a^	51.00 (7.50)^b^	*F*_(2,10)_ = 11.67; *P* = 0.002
δ^15^N	-3.28 (0.38)^ab^	-2.37 (0.32)^a^	-4.52 (0.44)^b^	*F*_(2,10)_ = 6.24; *P* = 0.017
δ^13^C (LTWUE)	-11.18 (0.56)^a^	-11.50 (0.29)^a^	-12.00 (0.09)^a^	*F*_(2,10)_ = 0.65; *P* = 0.541

*Different superscripts in each column indicate significant differences at *P* = 0.05.*

### Resource Use Efficiency Traits

**FIGURE 3:**
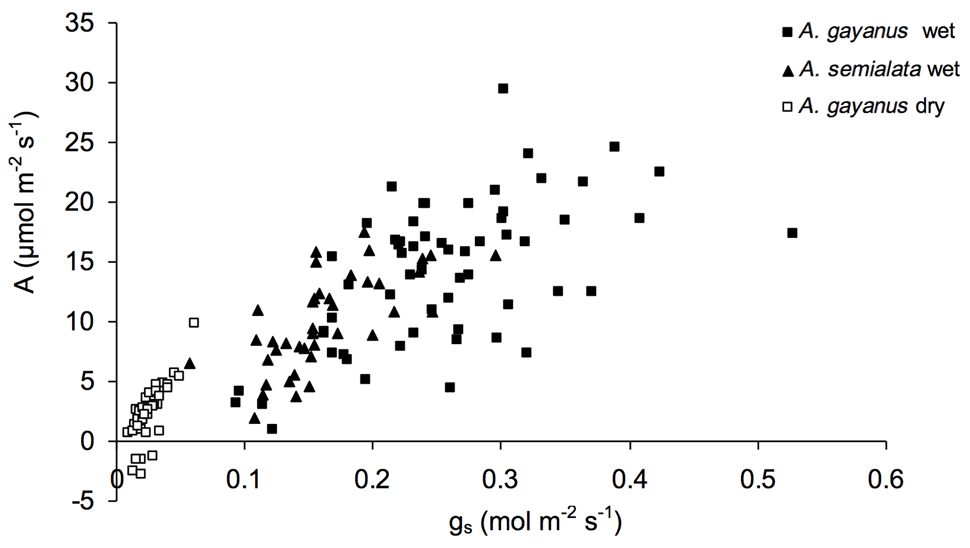
**Intrinsic water use efficiency (IWUE) of *A. gayanus* leaves in the wet (solid squares) and dry (open squares) seasons and *A. semialata* in the wet (triangles) seasons**.

**FIGURE 4:**
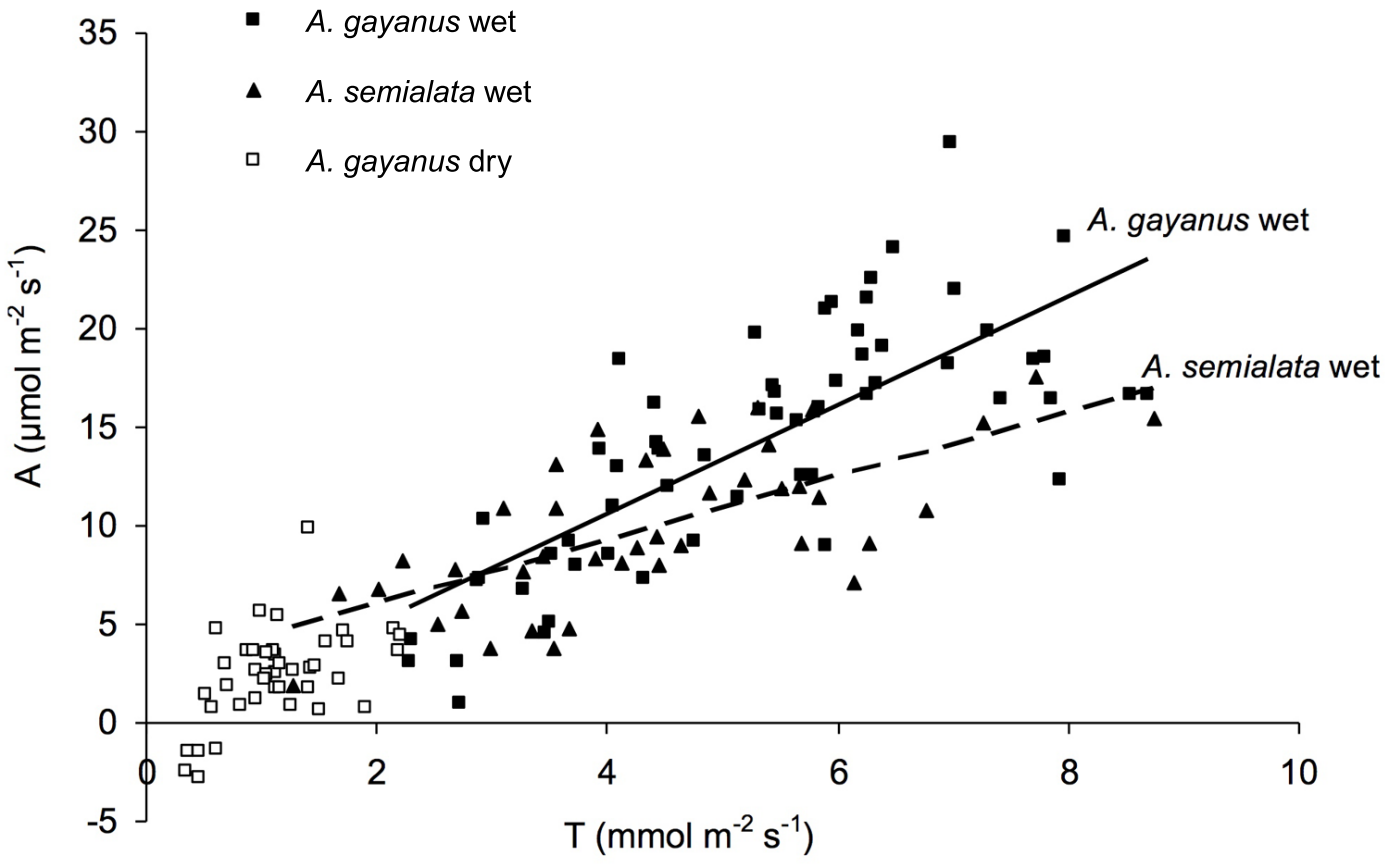
**Instantaneous transpiration efficiency (ITE) of *A. gayanus* leaves in the wet (solid squares and solid lines, *n* = 210), *A. semialata* in the wet (triangles and broken lines, *n* = 120), and *A. gayanus* in the dry (open squares, *n* = 120) seasons**.

**FIGURE 5:**
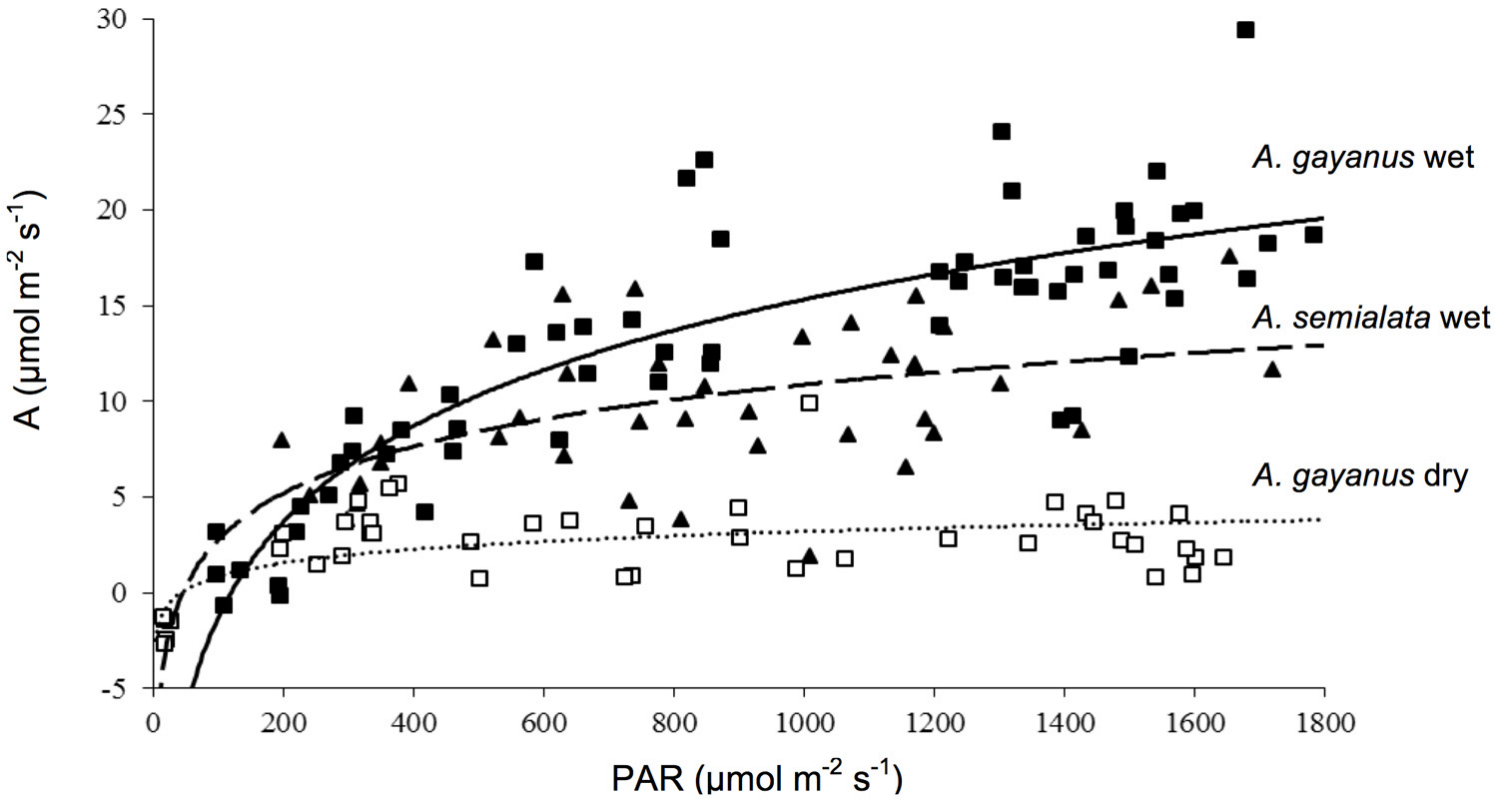
**Light response curves for leaves of *A. gayanus* (closed squares, solid line) and *A. semialata* (closed triangles, dashed line) in the wet season and *A. gayanus* in the dry season (open squares, dotted line)**.

*Andropogon gayanus* and *A. semialata* had similar water use efficiency (WUE) according to three different measurements: IWUE (**Figure 3**), LTWUE (δ^13^C, **Table 2**) and ITE (**Figure 4**; **Table 1**). The PNUE and A_max_, T_max_, g_s_ of *A. gayanus* in the wet season were significantly higher than *A. semialata* (**Table 1**), while there were no differences in LUE (**Figure 5**; **Table 1**). From the wet to dry seasons, *A. gayanus* leaves showed significant increases in IWUE and ITE (**Figure 4**; **Table 1**); however, there was no change in LTWUE (δ^13^C, **Table 2**). By the dry season, A_max_ of *A. gayanus* leaves had decreased by 82% (**Figure 5**; **Table 1**) relative to wet season rates, while PNUE was significantly higher (**Table 1**). The LUE of *A. gayanus* was significantly lower in the dry season compared to the wet season (**Table 1**; **Figure 5**).

## Discussion

Along with land use change and climate change, alien plant invasion is one of the most threatening processes for the maintenance of biodiversity and ecosystem function. Interdisciplinary research is clearly needed combining ecology, eco-physiology, hydrology, and invasion biology to better understand differences between native and invasive alien species that will assist management and restoration of invaded ecosystems (Gioria and Osborne, 2014). Both instantaneous and time-integrated resource use efficiency (RUE) measures are required to assess performance on short-term (seasonal) as well growth cycles and phases of invasion. In this study, we used comparative measures of both instantaneous (A, A_max_, T, T_max_, g_s_, ITE, LUE) and integrated measures of RUE (PNUE, LTWUE, LAI, biomass) to assess both resource acquisition and resource conservation performance in a highly seasonal environment.

Tropical savanna may represent a strong ‘habitat filter’ (after Weiher et al., 1998) as species grow and persist in a low N, annual drought affected, high water deficit, high VPD, and fire prone ecosystem. In such an environment, successful invasive species may exhibit similar resource conservation or RUE traits to native species that enable survival, however, this was not the case in this study. Under wet season conditions of high light, moisture, and N availability, rates of stomatal conductance, A and T of the alien species were 30–40% higher than the native species, with assimilation occurring for an additional 2 months of the year supported by a deep (up to 80 cm) and profusely branched, fibrous root mass (Rossiter-Rachor et al., 2009). Growth was maintained into the dry season with *A. gayanus* stand biomass and LAI exceeding that of the wet season (**Table 1**). This finding is consistent with the meta-analysis of Cavaleri and Sack (2010) who found that invaders typically have significantly higher rates of g_s_, water use and assimilation, although their analysis included few studies comparing invasive and native grasses.

While rates of A, T, and g_s_ were higher for the invader, there were no species differences in instantaneous water and light use efficiencies measures (IWUE, ITE, LTWUE, LUE), also consistent with meta-analysis of WUE of Cavaleri and Sack’s (2010), even when this analysis was restricted to arid and semi-arid ecosystems (Funk, 2013). While both species are perennial grasses, in this environment leaf function (and age) is essentially annual, with leaf initiation, development, and gas exchange occurring only after the onset of wet season rainfall. This is followed by senescence after seed set in March–April (native species) or May–June (*A. gayanus*). As a consequence, leaves of both species develop in high water availability and low LAVPD conditions with little difference in leaf-scale WUE. *A. gayanus* showed stomatal down-regulation and increases in IWUE and ITE (**Figures 3** and **4**) during the dry season, suggesting physiological plasticity in response to the higher LAVPD and reduced soil moisture availability. Physiological plasticity has been demonstrated for a number of invasive species compared to native species in low-resource environments where resource availability fluctuates (Funk, 2008; Davidson et al., 2011). This is a favorable attribute for persistence in the seasonal tropics, which are characterized by large seasonal changes in resource availability, in particular available N, P, and moisture (Hutley et al., 2000; Soper et al., 2015).

Differences in leaf scale traits alone were unable to explain the 5–10 times greater stand scale biomass accumulation and fourfold increase in LAI of the invader at these sites (**Table 1**). The exception was PNUE, reflecting one of the most significant limiting resource in these mesic savannas, soil available N (Rossiter-Rachor et al., 2009; Soper et al., 2015). Most studies comparing nutrient-use efficiency in native and alien plants have found higher PNUE in the invasive species (Funk, 2013). For example, the alien African lovegrass (*Eragrostis curvula*) had a higher PNUE compared to native grasses in the low-nutrient soils of eastern Australia (Firn et al., 2012). Plant invasion is thought to mostly occur in resource rich environments, with invasion driven by altered growing conditions and release of resources via disturbance that differentially increases an invader’s competitive attributes (Daehler, 2003). Recent evidence suggests that invasion and persistence does occur in low resource environments; however, drivers of this are poorly understood (Gioria and Osborne, 2014).

The invasive traits of *A. gayanus* identified in this study exhibit all three attributes suggested by Funk and Vitousek (2007) that are critical for invasion and perseverance in low-resource environments; (1) high resource acquisition and high RUE, (2) an active increase in resource availability following invasion, and (3) continued disturbance following invasion. Firstly, resource acquisition and RUE were exhibited by *A. gayanus* via higher rates of g_s_, A, and T, a longer growing season, high biomass and LAI and significantly higher PNUE. Secondly, an invasive species must actively increase resource availability. Comparative values of leaf δ^15^N (**Table 2**) suggested *A. gayanus* is likely to use more soil ammonium or have increase soil ammonium levels when compared to native grass dominated patches. This is consistent with findings of Rossiter-Rachor et al. (2009) who used labeled ^15^N experiments that showed *A. gayanus’* preference for ammonium as an N source over nitrate. The presence of *A. gayanus* stimulated soil ammonification and potentially inhibited nitrification (Rossiter-Rachor et al., 2009). This mechanism drives a positive plant–soil feedback that promotes a broader niche width and improved habitat suitability for *A. gayanus*, in this N-limited ecosystem. Thirdly, Funk and Vitousek (2007) suggest an invader must promote continued disturbance that increases resource availability enabling persistence. A feature of *A. gayanus* invasion is high biomass (fuel) production and a shift to a high severity fire regime as described by Rossiter et al. (2003) and Setterfield et al. (2010). Severe invasion reduces woody cover by up to 80% within a decade post invasion (Brooks et al., 2010) and initiates a grass-fire feedback. The loss in woody cover releases water, nutrient resources and increases radiation to the understory that further accelerates *A. gayanus* growth and invasion.

## Conclusion

This study has shown that collectively, instantaneous, and time-integrated RUE traits, invasion-derived feedback loops combined with high propagule pressure confers substantial a competitive advantage to *A. gayanus* over both native grassy and woody lifeforms. These attributes largely explain its current invasiveness and persistence in Australia’s low-resource savanna ecosystems. This is an ecosystem transformation that is resulting in a rapid loss of biodiversity and significantly increasing fire risk (Setterfield et al., 2013). This study provides evidence from a seasonal tropical savanna ecosystem to support Funk’s (2013) assertion that invasive species in low-resource environments possess traits that allow both increased resource acquisition and resource conservation. This superior capacity of *A. gayanus* to compete for resources also supports modeling predictions of continued rapid invasion across the vast savanna region of northern Australia (Adams et al., 2015).

## Conflict of Interest Statement

The authors declare that the research was conducted in the absence of any commercial or financial relationships that could be construed as a potential conflict of interest.
